# Comparison of the Effect of Unfractionated Heparin and Enoxaparin Sodium at Different Doses on the Course of COVID-19-Associated Coagulopathy

**DOI:** 10.3390/life11101032

**Published:** 2021-09-30

**Authors:** Oleksandr Oliynyk, Wojciech Barg, Anna Slifirczyk, Yanina Oliynyk, Serhij Dubrov, Vitaliy Gurianov, Marta Rorat

**Affiliations:** 1 Department of Anaesthesiology and Intensive Care, Bogomolets National Medical University, 01601 Kyiv, Ukraine; alexanderoliynyk8@gmail.com (O.O.); president@aaukr.org (S.D.); 2 Department of Emergency Medicine, Pope John II State School of Higher Education in Biala Podlaska, 21-500 Biala Podlaska, Poland; rektor@pswbp.pl; 3 Department of Internal Medicine, Pneumonology and Allergology, Wroclaw Medical University, 50-367 Wroclaw, Poland; wojciech.barg@umed.wroc.pl; 4 Department of Immunology and Allergology, Bogomolets National Medical University, 01601 Kyiv, Ukraine; kafedra.imun@gmail.com; 5 Department of Medical Statistics, Bogomolets National Medical University, 01601 Kyiv, Ukraine; managementnmu@gmail.com; 6 Department of Forensic Medicine, Wroclaw Medical University, 50-367 Wroclaw, Poland

**Keywords:** anticoagulant therapy, blood coagulation, SARS-CoV-2, respiratory failure

## Abstract

Background: COVID-19-associated coagulopathy (CAC) exacerbates the course of coronavirus infection and contributes to increased mortality. Current recommendations for CAC treatment include the use of low-molecular weight heparins (LMWH) at prophylactic or therapeutic doses, as well as the use of unfractionated heparin (UFH). Methods: A randomised, controlled trial enrolled 126 patients hospitalised in the intensive care unit with severe COVID-19 complicated by CAC. The effects of LMWH at preventive and therapeutic doses and UFH at therapeutic doses on mortality and intubation rates were compared. Results: The number of intubations and deaths showed no significant difference depending on the anticoagulant therapy used. However, multivariate logistic regression models revealed an increased risk of intubation (*p* = 0.026, odds ratio (OR) = 3.33, 95% confidence interval (CI) 1.15–9.59), and an increased risk of death (*p* = 0.046, OR = 3.01, 95% CI 1.02–8.90), for patients treated with LMWH at a prophylactic dose but not at a therapeutic dose as compared to patients treated with UFH when controlling for other risk factors. Conclusions: The use of unfractionated heparin in the treatment of COVID-19-associated coagulopathy seems to be more effective at reducing the risk of intubation and death than enoxaparin at prophylactic doses.

## 1. Introduction

The impact of coagulation disorders in the course of COVID-19 (coronavirus disease 2019) has been proven. COVID-19 is a pronounced prothrombotic condition resulting from the dysregulation of the coagulation cascade [1,2]. The pro-inflammatory activation, known as cytokine release syndrome, which arises during the development of SARS-CoV-2 (severe acute respiratory syndrome coronavirus 2) activates the coagulation cascade, leading to thrombosis. Just like in severe sepsis, the generalized deposition of intravascular clots disrupts the blood supply to the internal organs, leading to organ failure and death [3,4]. There is evidence of the direct introduction of the SARS-CoV-2 virus into endothelial cells, which can potentially lead to their damage. It has been suggested that endothelial damage, microvascular inflammation, endothelial exocytosis, and/or endotheliitis play a major role in the pathogenesis of acute respiratory distress in patients with severe COVID-19 [5,6,7]. The mechanism of hypercoagulability in patients with COVID-19 is presumably associated with severe endothelial dysfunction and the induction of platelet aggregation (the endothelium carries ACE2 receptors) [8]. It is also known that in patients with severe COVID-19, changes in several circulating prothrombotic factors appear: an increased level of factor VIII, an increased level of fibrinogen, and the circulation of prothrombotic microparticles [9,10]. The resulting blood coagulation disorders are considered to be COVID-19-associated coagulopathy (CAC) [11]. To some extent, coagulopathy symptoms occur in all patients with severe COVID-19 [12]. In patients with coagulopathy, thromboprophylaxis with low-molecular-weight heparins (LMWH) is recommended to prevent thromboembolic events [13,14]. In general, there are prophylactic, intermediate, and therapeutic doses of LMWH. Most authors consider the prophylactic dose to be 50 anti-Xa IU/kg once a day (QD), the intermediate dose to be 100 anti-Xa IU/kg QD, and the therapeutic dose to be 100 anti-Xa IU/kg twice a day (BID) [15,16]. Such treatment reliably reduces mortality [17]. It is often the case that despite the use of preventive doses of LMWH, laboratory coagulation parameters like the prothrombin index, activated partial thromboplastin time (APTT), and serum fibrinogen confirm ongoing hypercoagulation. This proves that coagulopathy in those patients cannot be corrected with preventive doses of LMWH. Some experts believe that therapeutic doses of LMWH or unfractionated heparin (UFH) should be used in such cases [8,18]. However, the use of therapeutic doses of LMWH or UFH for the treatment of coagulopathy is still subject to debate. The existing COVID-19 treatment protocols declare that studies comparing the pharmacological effects of different anticoagulants at prophylactic and therapeutic doses are highly encouraged [15,19]. Determining the best antithrombotic agent for CAC and its optimal dose are of crucial importance to the successful treatment of COVID-19. This study aimed to compare the impact of LMWH at prophylactic and therapeutic doses and UFH at a therapeutic dose on mortality and intubation rates in patients with severe COVID-19 and associated coagulopathy.

## 2. Materials and Methods

A randomised, controlled trial enrolled 126 patients with severe COVID-19 and CAC, who were hospitalized at the Department of Anaesthesiology and Intensive Care for infectious patients at the Kyiv City Clinical Hospital No.4 from 1 July 2020 to 1 March 2021.

Inclusion criteria comprised:

1. confirmed SARS-CoV-2 infection (confirmed with a positive reverse transcription polymerase chain reaction (RT PCR) test),
2. the presence of bilateral interstitial pneumonia on a computed tomography (CT) scan,
3. respiratory failure with arterial partial pressure of oxygen (PaO_2_) < 60 mm Hg with room air,
4. a D-dimer level > 3 mg/L,
5. a platelet count < 120 × 10^9^/L, and
6. the informed consent of the patient or their legal representative to participate in the study.

Exclusion criteria comprised:

1. respiratory failure requiring intubation prior to enrolment,
2. hypersensitivity to enoxaparin sodium or unfractionated heparin,
3. a history of heparin-induced type-II thrombocytopenia caused by the use of unfractionated heparin or low-molecular-weight heparin,
4. clinically significant active bleeding,
5. creatinine clearance (Cockcroft-Gault formula) < 30 mL/min,
6. unstable arterial hypertension (systolic pressure > 180 mmHg or diastolic pressure > 110 mmHg),
7. pulmonary embolism (PE) confirmed by a CT pulmonary angiography,
8. pregnancy or breastfeeding, and
9. participation in other clinical studies.

All patients who met the inclusion criteria and presented no exclusion criteria were randomly split into three groups. Forty-two patients in each group received:

Group 1: LMWH—enoxaparin (E, Flenox by Farmak, Kyiv, Ukraine) at a preventive (prev) dose of 50 anti-Xa IU/kg QD subcutaneously;

Group 2: LMWH—enoxaparin at a therapeutic (ther) dose of 100 anti-Xa IU/kg BID subcutaneously;

Group 3: UFH—heparin sodium at an initial dose of 80 U/kg/h intravenously, followed by a maintenance dose of 18 U/kg/h (hereinafter referred to as UFH-ther). The dose of UFH was adjusted based on APTT which was kept within the range of 40–70 s. 

Therapy with LMWH-ther and UFH was continued until the D-dimer values normalized. The medical report comprised the patient’s medical history, physical examination, and laboratory tests: complete blood count, arterial blood gases, C-reactive protein (CRP), procalcitonin (PCT), fibrinogen, D-dimer, Il-6, and ferritin. The research compared the effect of the heparins used on 28-day mortality and intubation rates.

### Statistical Analysis

MedCalc^®^ Statistical Software version 19.5.6 (MedCalc Software Ltd., Ostend, Belgium; https://www.medcalc.org; 2020, accessed on 20 May 2021) was used in the analysis. As the law of distribution differed from normal by the Shapiro-Wilk test, the median (Me) and interquartile range (QI–QIII) were calculated to represent quantitative data. Frequency (%) was calculated for qualitative measures. The Kruskal-Wallis test was used to compare quantitative characteristics in the three groups, and subsequent comparisons were made using the Dunn test. To compare qualitative characteristics in more than two groups, a chi-squared test was used, and subsequent comparisons were made using Fisher’s exact test with Bonferroni correction taken into account. Fisher’s exact test was used to compare frequencies between the two groups. To quantify the degree of influence of characteristic factors on the risk of death, we used the method of constructing and analysing logistic regression models. The prognostic quality of the models was assessed using the method of building the receiver operating characteristic curves (ROC curve). The area under the ROC curve (AUC) and its 95% CI were calculated. The effect of the factors was assessed by the value of the odds ratios (OR), for which a 95% CI was calculated. For all statistical tests, the *p* value < 0.05 was considered significant.

## 3. Results

Demographic, clinical, and laboratory characteristics of the study groups are shown in Table 1. All the patients had an increased risk of venous thromboembolism according to the Padua prediction score (PPS) ≥ 4 [20]. Recently, this tool has also been validated for COVID-19 patients [21]. As shown in Table 1, the median values of the investigated parameters did not differ significantly between the groups. The exceptions were the medians of white blood cell count, respiratory index, and heart rate.

**Figure 1:**
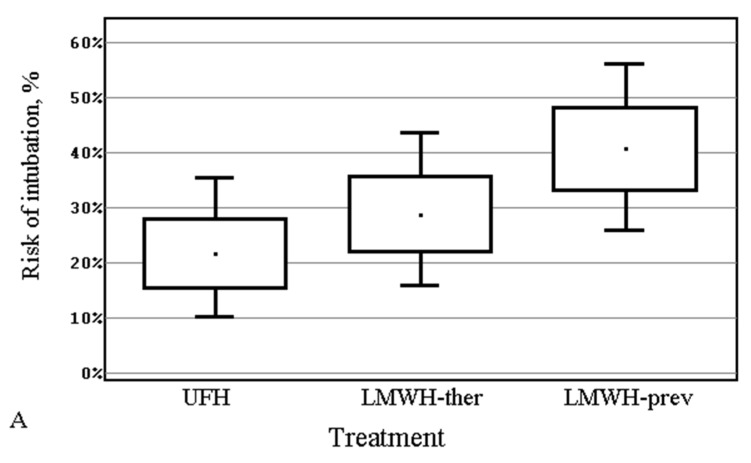

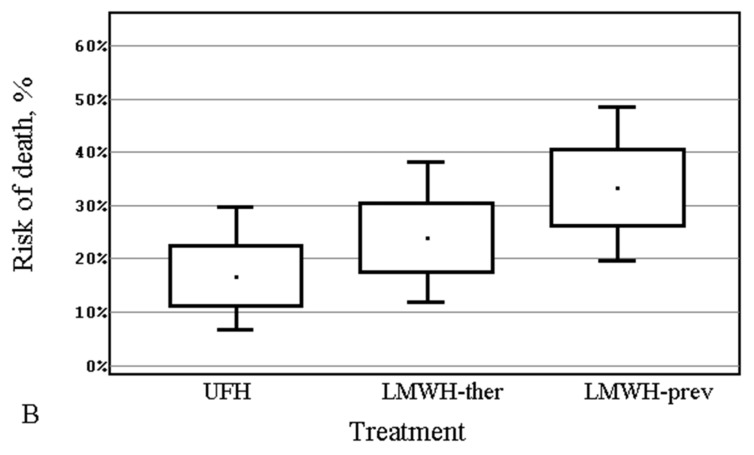
Risk and 95% CI of intubation (**A**) and death (**B**) in patients treated with unfractionated heparin (UFH) or low-molecular-weight heparin (LMWH) either at therapeutic (ther) or preventive (prev doses).

During the 28-day observation period, the intubation rates were 17, 10, and 9 patients while the mortality rates were 14, 10, and 7 for LMWH-prev, LMWH-ther, and UFH groups, respectively (Figure 1). None of the patients were intubated at the time of admission to the intensive care unit (ICU) and before the initiation of heparin therapy. Figure 1 shows the risk of intubation (1A) and death (1B). There were no statistically significant differences between the groups either for intubation or for death (chi-squared test, *p* = 0.156, and *p* = 0.205, respectively). Positive trends in the number of intubations and deaths were observed when comparing UFH therapy to both LMWH therapies. Positive trends were also seen if therapeutic doses of LMWH were compared to LMWH at prophylactic doses.

**Figure 2:**
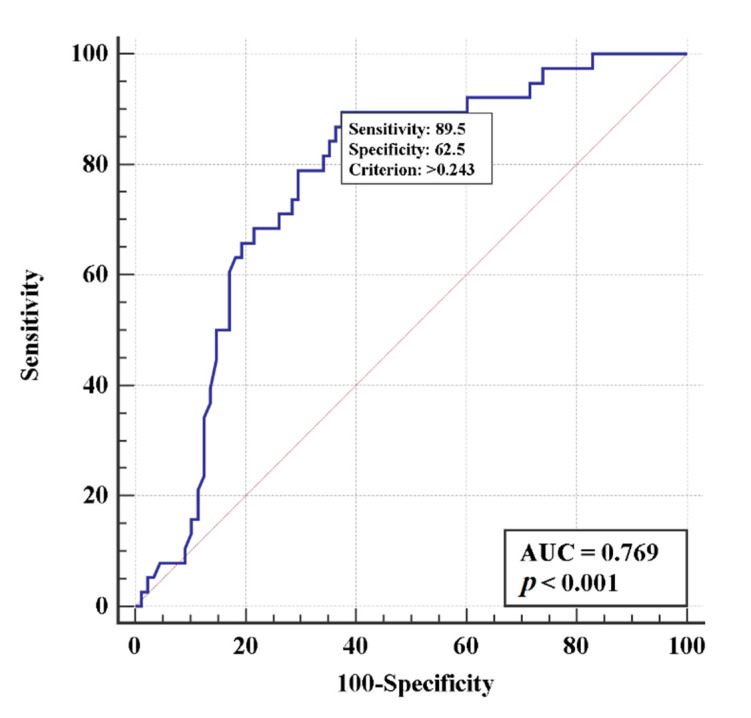
Multivariate logistic regression models using D-Dimer levels, sex, and the type of treatment to estimate the risk of intubation.

**Figure 3:**
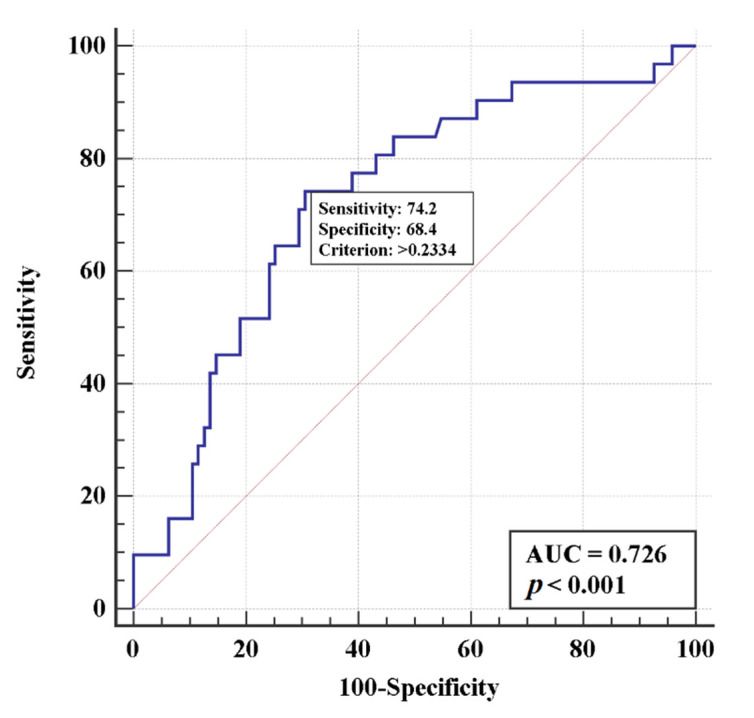
Multivariate logistic regression models using D-Dimer levels, age, and the type of treatment to estimate the risk of death.

A multivariate logistic regression modelling method was used to identify risk factors for intubation or death. Three factors associated with increased risks of both intubation and death were selected: D-dimer, sex or age, and the type of treatment. The model based on these features (Figure 2 and Figure 3) is adequate: AUCs were 0.77 (95% CI 0.69–0.84) and 0.73 (95% CI 0.64–0.80) for intubation and death risks, respectively, indicating a moderate relationship between the determined factors and the risks of intubation or death.

The results of the intubation and death risks analyses according to the chosen models are shown in Table 2 and Table 3, respectively.

There were increased risks of intubation (*p* = 0.026, OR = 3.33; 95% CI 1.15–9.59) and death (*p* = 0.046, OR = 3.01; 95% CI 1.02–8.90), respectively, for patients treated with LMWH at the prophylactic dose, as compared to patients treated with UFH when controlling for the two other risk factors, i.e., D-dimer concentration and sex (intubation) or age (death). Thus, whilst the univariate analysis of the data showed no significant effect of the different anticoagulant treatments on the number of intubations and deaths, the use of multivariate logistic regression models proved the advantage of UFH over LMWH at a prophylactic dose.

Two patients in the UFH group developed thrombocytopenia <50.0 × 10^9^/L and two others harmless bleeding: one a subcutaneous haemorrhage in an upper limb, another in the abdominal wall. In these cases, treatment with UFH was stopped, the patients were switched to LMWH, and withdrawn from the study. Both patients survived. In the two other groups studied, there were no complications observed. There was no statistically significant difference in the risk of thrombocytopenia and bleeding between the groups (chi-squared test, *p* = 0.131 in both cases).

## 4. Discussion

The etiology of CAC is multifactorial. It includes activated coagulation, endotheliopathy, up-regulated innate and adaptive immunity, as well as an activated complement system [22,23]. However, hypoxia itself seems to be a key factor. Alveoli type II epithelial cells suffering from hypoxia stimulate coagulation cascades. This leads to the formation of microthrombi and aggravates hypoxia, causing a vicious cycle, ultimately resulting in irreversible lung damage [24]. In addition, viral replication causes infiltration of inflammatory cells, triggering coagulation cascades, the development of a cytokine storm, endothelial cell apoptosis, and microvascular thrombosis [25]. Interleukin-6 levels have been found to correlate positively with fibrinogen levels, confirming the link between inflammation and procoagulant changes [24].

Currently, there are no clear diagnostic criteria for CAC. This syndrome is associated with abnormal test results of the coagulation system but does not correspond to classic signs of coagulopathy. Due to numerous similarities, it is considered a thrombotic phenotype of DIC [23]. Ibaet al. proposed their own definition of CAC. It follows that the patients have to meet at least two out of four criteria: (1) a decrease in platelet count (<150 × 10^9^/L); (2) an increase in D-dimer levels (>2 × the upper limit of normal); (3) >1 s prolonged prothrombin time or INR >1.2; (4) the presence of thrombosis (macrothrombosis and/or microthrombosis, including skin and acral lesions, etc.) [23]. According to other researchers, CAC can be diagnosed in patients with confirmed or suspected COVID-19 if the following criteria are present: a platelet count <120 × 10^9^/L, a decrease in APTT below normal values, and increased fibrinogen and D-dimer concentrations without clinical evidence of primary diseases of the blood system or liver [3,26]. A D-dimer concentration above 1.5 mg/mL can be used as a marker of high risk for CAC [11]. Coagulopathy is one of the most frequent pathologies in patients who die due to COVID-19. It was diagnosed in 71.4% of deceased patients [27]. Consequently, it is considered as one of the most significant prognostically unfavourable features in patients with COVID-19 [28].

COVID-19 mortality directly correlates with blood D-dimer levels and the magnitude of the decrease in serum platelet count [12]. Compared to non-COVID-19 patients, COVID-19 patients have a significantly higher probability of pulmonary vascular thrombosis [14,25]. Considering that autopsy data from patients with COVID-19 show extensive microthrombi in alveoli, myocardium, and renal tubular epithelia, anticoagulant therapy seems to be essential [11]. LMWH and UFH are recommended for the treatment of coagulopathy, although LMWH is currently preferred [14]. In critically ill patients, anticoagulant therapy with heparins at intermediate or therapeutic doses was associated with a lower probability of pulmonary embolism (OR 0.09; 95% CI, 0.02–0.57) but a higher probability of major bleeding (OR 3.84; 95% CI, 1.44–10.21) [29]. There is insufficient evidence to recommend or reject the use of anticoagulants at therapeutic doses to prevent thrombotic complications in hospitalised patients with COVID-19 [15]. There is also no conclusive evidence that any particular heparin formulation has an advantage in the treatment of COVID-19 and will affect disease outcomes. There is also no clear opinion regarding the doses of heparin in patients hospitalised with COVID-19 [30]. The majority of researchers tend to use standard prophylactic doses, but some prefer higher (intermediate or therapeutic) doses. Among 46 experts from an international working group, 31.6% were in favour of the use of intermediate doses in patients without DIC, and 5.2% were in favour of therapeutic doses [30]. Current guidelines for the treatment of COVID-19 welcome randomised trials to compare the effectiveness of anticoagulants at different doses [15,19].

In patients with blood D-dimer levels above 3.0 mg/L who received LMWH, the mortality rate was 32.8%, while in those who did not receive LMWH it was 52.4%, *p* = 0.017 [31]. It is concluded that anticoagulant therapy with LMWH is associated with a better prognosis in patients with severe COVID-19 and elevated D-dimers. According to Lucatelli et al., the 28-day mortality rate among patients with COVID-19 receiving LMWH was 40.0%, as compared to 64.2% in those not receiving LMWH, (*p* = 0.029) [32]. Cohen et al. observed advantages of LMWH over UFH because of an observed reduction in the incidence of major bleeding and lower overall mortality, but the study results were statistically insignificant [33]. Due to insufficient evidence, most guidelines do not indicate which heparin should be used in preventing thrombotic events [14,34,35]. A meta-analysis of 23 comparative studies of LMWH and UFH in 9587 patients with various diseases (non-COVID-19) showed that deaths and major bleeding were less frequent when using LMWH. However, it was concluded that the authors of the publications analysed were prejudiced against UFH and that these studies were methodologically flawed [36].

The biggest advantage of LMWH over UFH is the ability to use subcutaneous injections of fixed doses of LMWH per kg of body weight, without continuous monitoring of the coagulation and continuous dosage adjustments. The use of LMWH allows patients to receive treatment at home rather than in hospitals [37]. However, UFH is the drug of choice in the case of concomitant renal failure (with creatinine clearance below 30 mL/min) or unstable hemodynamics [34].

Our study revealed a downward trend in the number of intubations and mortality in patients receiving UFH as compared to LMWH. In our opinion, this can be explained by the different anticoagulant properties of the drugs. UFH can affect the thrombus by enhancing thrombolysis, as UFH in PE is known to restore impaired pulmonary perfusion [34]. At least half of UFH molecules bind with high affinity to antithrombin [38] and accelerate the inhibition of thrombin as well as factors Xa, IXa, XIa, and XIIa. The UFH changes the antithrombin molecule, which makes interaction with the protease targets much easier. It is known that UFH and LMWH have anti-inflammatory and antiviral effects [22]. The strength of these effects may vary. UFH inactivates the SARS-CoV-2 virus and prevents its entry into mammalian cells, thereby inhibiting the entry of the virus into lung tissue [39]. LMWH and UFH molecules bind to the spike protein, resulting in the suppression of viral infection in cells [17].

To date, studies comparing the therapeutic effects of UFH and LMWH at different doses are infrequent. A large randomised NIAID trial [40] is currently underway, bringing together three clinical trial platforms covering four continents and over 300 hospitals. The study includes REMAP-CAP, ACTIV-4, and ATTACC studies and over 1000 hospitalised COVID-19 patients. The very preliminary results suggest that therapeutic doses of LMWH are no less effective than preventive ones at improving treatment outcomes in patients with COVID-19 [40]. Researchers suggest that therapeutic doses of anticoagulants are not only safe but also superior to preventive ones. The US National Institutes of Health stated that with a large number of patients with COVID-19 requiring hospitalisation, the results of this study could help reduce the burden on ICUs around the world. Researchers are currently working to provide the full results of the data. Our findings seem to be consistent with the interim results of this large but not yet fully completed study.

## 5. Limitations

A weak statistical basis, which may be the result of a low number of patients in the study groups, significantly limits the power of this study. Statistically significant differences between the groups were found only as a result of the use of multivariate logistic regression models.

## 6. Conclusions

Based on the univariate analysis, our study revealed that the use of unfractionated heparin in the treatment of COVID-19-associated coagulopathy at a therapeutic dose was no less effective than the use of enoxaparin at prophylactic and therapeutic doses in reducing the number of intubations and patient mortality. However, the use of multivariate logistic regression models has proved the advantage of unfractionated heparin over enoxaparin in reducing the risk of intubation and death at a prophylactic dose but not at a therapeutic one. The number of bleeding complications with unfractionated heparin is not significantly different from that with enoxaparin. There is a need for further research on a larger group of patients.

## Figures and Tables

**Table 1:** Demographic, clinical, and laboratory characteristics of the study groups, median (QI–QIII).

	LMWH-Prev (*n* = 42)	LMWH-Ther (*n* = 42)	UFH (*n* = 42)	*p* Value
Age, years	71 (68–72)	70 (68–72)	71 (69–72)	0.525
Female (%)	15 (35.7)	16 (38.1)	19 (45.2)	0.650
Temperature, °C	37.5 (37.4–37.9)	37.5 (37.4–37.8)	37.5 (37.3–37.7)	0.712
Heart rate, beat per min	83.5 ** (80–86)	86 (84–88)	86 (84–88)	**0.018**
CRP, mg/L	48 (34–72)	44.5 (35–66)	50 (39–72)	0.480
Interleukin-6, pg/mL	34 (32–45)	33.5 (23–60)	36.5 (33–44)	0.757
Procalcitonin, ng/mL	0.6 (0.5–0.8)	0.6 (0.6–0.7)	0.6 (0.4–0.8)	0.321
Ferritin, ng/mL	443 (365–556)	450.5 (345–554)	446 (348–545)	0.997
Fibrinogen, g/L	6.4 (5.5–6.5)	5.6 (5.5–5.9)	5.5 (5.4–5.9)	0.215
D-dimer, µg/L	5246 (3567–5657)	4494 (4221–5664)	5245 (4221–5445)	0.853
Leukocytes, × 10^9^/L	4.2 ** (4.0–4.3)	4.7 * (4.1–4.8)	4.2 (3.5–4.3)	**0.008**
Lymphocytes, %	23.5 (22–26)	18 (17–24)	24 (22–26)	0.053
Thrombocytes, × 10^9^/L	127 (122–138)	128 (102–165)	130 (126–143)	0.379
Erythrocytes, × 10^12^/L	2.8 (2.5–3.3)	2.7 (2.3–3.4)	2.8 (2.6–3.3)	0.498
PaO_2_/FiO_2_, mm Hg	145 ** (124–176)	124 * (119–139)	147 (132–176)	**0.039**

Laboratory test reference ranges: CRP < 5.0 mg/L, interleukin-6 < 4.0 pg/mL, procalcitonin < 0.02 ng/mL, ferritin 8–143 ng/mL, fibrinogen 2.0–4.0 g/L, D-dimer < 500 µg/L, leukocytes 4.0–9.0 × 10^9^/L, lymphocytes 19–37%, thrombocytes 200–400 × 10^9^/L, erythrocytes 3.6–4.2 × 10^12^/L, PaO_2_/FiO_2_ 454–495 mm Hg. LMWH-prev—enoxaparin, prophylactic dose; LMWH-ther—enoxaparin, therapeutic dose; UFH—unfractionated heparin. *—statistically significant difference from the group of patients treated with UFH, *p* < 0.05. **— statistically significant difference from the group of patients treated with enoxaparin at the therapeutic dose, *p* < 0.05.

**Table 2:** Analysis of risk of intubation in a three-factor logistic regression model.

Factorial Feature		The Model Coefficient, b ± m	Significance Level of Difference of the Coefficient from 0, *p* Value	OR (95% CI)
Treatment	UFH therapeutic dose	Reference		
	LMWH, therapeutic dose	0.56 ± 0.55	0.308	-
	LMWH, prophylactic dose	1.20 ± 0.54	**0.026**	3.33 (1.15–9.59)
Sex		−0.81 ± 0.43	0.061	0.44 (0.19–1.04)
D-dimer, µg/L		0.0008 ± 0.0002	**0.001**	1.001 (1.000–1.001)

**Table 3:** Analysis of risk of death in a three-factor logistic regression model.

Factorial Feature		The Model Coefficient, b ± m	Significance Level of Difference of the Coefficient from 0, *p* Value	OR (95% CI)
Treatment	UFH therapeutic dose	Reference		
	LMWH, therapeutic dose	0.59 ± 0.58	0.311	-
	LMWH, prophylactic dose	1.10 ± 0.55	**0.046**	3.01 (1.02–8.90)
Age, years		0.12 ± 0.05	0.030	1.13 (1.01–1.25)
D-dimer, µg/L		0.0006 ± 0.0002	**0.017**	1.001 (1.000–1.001)

## Data Availability

The data presented in this study are available on request from the corresponding author. The data are not publicly available due to ethical aspects.
